# Validation of sorghum quality control (QC) markers across African breeding lines

**DOI:** 10.1002/tpg2.20438

**Published:** 2024-02-26

**Authors:** Davis M. Gimode, Grace Ochieng, Santosh Deshpande, Eric O. Manyasa, Clarisse P. Kondombo, Erick O. Mikwa, Millicent O. Avosa, Josephine Sarah Kunguni, Kahiu Ngugi, Patrick Sheunda, McDonald Bright Jumbo, Damaris A. Odeny

**Affiliations:** ^1^ International Crops Research Institute for the Semi‐Arid Tropics Nairobi Kenya; ^2^ International Crops Research Institute for the Semi‐arid Tropics‐Patancheru Patancheru Telangana India; ^3^ Institut de l'Environnement et de Recherches Agricoles (INERA) Ouagadougou Burkina Faso; ^4^ Department of Plant Breeding, IFZ Research Centre for Biosystems, Land Use and Nutrition Justus Liebig University Giessen Germany; ^5^ Department of Plant Science & Crop Protection University of Nairobi Nairobi Kenya; ^6^ The Kenya Seed Company Limited, Kitale Branch Kitale Kenya; ^7^ International Crops Research Institute for the Semi‐Arid Tropics (ICRISAT) Bamako Mali

## Abstract

Sorghum [*Sorghum bicolor* (L.) Moench] is a cereal crop of critical importance in the semi‐arid tropics, particularly in Africa where it is second only to maize (*Zea mays* L.) by area of cultivation. The International Crops Research Institute for the Semi‐Arid Tropics sorghum breeding program for Eastern and Southern Africa is the largest in the region and develops improved varieties for target agro‐ecologies. Varietal purity and correct confirmation of new crosses are essential for the integrity and efficiency of a breeding program. We used 49 quality control (QC) kompetitive allele‐specific PCR single nucleotide polymorphism (SNP) markers to genotype 716 breeding lines. Note that 46 SNPs were polymorphic with the top 10 most informative revealing polymorphism information content (PIC), minor allele frequency (MAF), and observed heterozygosity (*H*_o_) of 0.37, 0.43, and 0.02, respectively, and explaining 45% of genetic variance within the first two principal components (PC). Thirty‐nine markers were highly informative across 16 Burkina Faso breeding lines, out of which the top 10 revealed average PIC, MAF, and *H*_o_ of 0.36, 0.39, and 0.05, respectively. Discriminant analysis of principal components done using top 30 markers separated the breeding lines into five major clusters, three of which were distinct. Six of the top 10 most informative markers successfully confirmed hybridization of crosses between genotypes IESV240, KARIMTAMA1, F6YQ212, and FRAMIDA. A set of 10, 20, and 30 most informative markers are recommended for routine QC applications. Future effort should focus on the deployment of these markers in breeding programs for enhanced genetic gain.

## Core Ideas

- Validated informative single nucleotide polymorphism markers should be deployed for routine use in African breeding programs.
- The use of quality control markers for routine hybridity confirmation will fast track varietal development process.
- The ease of using kompetitive allele‐specific PCR markers will enhance their utilization in sorghum breeding programs.

**Abbreviations**
BIC: Bayesian information criterion
DAPC: discriminant analysis of principal component
DL: dry lowland
DT: drought tolerant
ESA: Eastern and Southern Africa
ICRISAT: The International Crops Research Institute for the Semi‐Arid Tropics
KASP: kompetitive allele‐specific PCR
LT: low temperature
MAF: minor allele frequency
PCA: principal components analysis
PIC: polymorphism information content
QC: quality control
SH: sub humid
SNP: single nucleotide polymorphism

## 1 INTRODUCTION

Sorghum ([*Sorghum bicolor* (L.) Moench], 2*n* = 2*x* = 20) is an important cereal staple crop that is mostly grown by smallholder farmers in the semi‐arid regions of the world. Sorghum production is ranked fifth globally at 40 million ha and only second to maize (*Zea mays* L.) in Africa (FAOSTAT, 2022). Due to its higher tolerance to various abiotic stresses, including drought and heat (Hao et al., 2021), sorghum plays a critical role in contributing toward food security in marginal environments. Among several uses for sorghum is food, feed, forage, fuel, beverage, and building material (Paterson et al., 2009). Sorghum grain is gluten free and rich in antioxidants and protein, which makes it a healthy alternative for baking (de Oliveira et al., 2022). It is believed to have been domesticated in Ethiopia and surrounding countries, from where it spread through human migration and trade to other regions of Africa, the Middle East, India, and the Far East (Dillon et al., 2007; Doggett, 1991; Fuller & Stevens, 2018).

Tremendous genetic variation is inherent in sorghum, which, coupled with its spread, has enabled wide adaptation to various agroecologies, climates, and environments. The five racial groups within cultivated sorghum are distinct morphologically (de Wet & Huckabay, 1967; Harlan & de Wet, 1972), which include bicolor, guinea, caudatum, kafir, and durra. The high genetic variation in the sorghum gene pool is an important genetic repository for crop improvement. For example, significant genetic variation has been reported for grain yield (Jordan et al., 2011; Mengistu et al., 2020) and quality, resistance/tolerance to biotic stresses such as anthracnose (*Colletotrichum sublineolum*) (Mengistu et al., 2020) and Striga (*Striga hermonthica*) (Mallu et al., 2021), tolerance to abiotic stresses including drought (Ochieng et al., 2020), heat (Chopra et al., 2017), cold (Ortiz et al., 2017), and salinity (Hao et al., 2021; Upadhyaya et al., 2019).

The International Crops Research Institute for the Semi‐Arid Tropics (ICRISAT) sorghum breeding program in Eastern and Southern Africa (ESA) has been leveraging on the abundant genetic diversity toward the improvement of sorghum varieties and development of hybrids for five major market segments. The same program also generates sorghum genetic stock that supports the national breeding programs in ESA. For effective utilization of the breeding material in the ICRISAT‐ESA program, an understanding of the level of genetic variation across breeding lines and novel germplasm is essential. Single nucleotide polymorphism (SNP) markers are currently the markers of choice and have been successfully used in different sorghum studies, including in the characterization of accessions from multiple institutes in West Africa (Afolayan et al., 2019), Ethiopian sorghum landraces (Girma et al., 2020; Menamo et al., 2021; Wondimu et al., 2021), core sets of germplasm from Ethiopia and Sudan in the USDA‐NPGS (Cuevas & Prom, 2020; Cuevas et al., 2017), and for sorghum germplasm across agroclimatic zones in Niger (Maina et al., 2018).

Although SNPs are highly informative and abundant in crop genomes, they can be costly to run, if not well optimized for targeted breeding applications. The recent recommendation of developing a set of cost‐effective, optimized, and highly informative markers for routine use in breeding programs for quality control (QC) is an excellent concept for wide application in sorghum breeding programs. Informative QC markers can be used for fingerprinting of elite lines and released varieties, hybridity confirmation, and selection of diverse genotypes for new crossing blocks. In sorghum, a set of 49 Kompetitive allele‐specific PCR (KASP) QC markers have been developed and made accessible at Intertek‐Agritech (https://www.intertek.com/agriculture/agritech/) through the Excellence in Breeding Platform (https://excellenceinbreeding.org/). The objective of our study was to validate the informativeness of these QC markers for ESA sorghum breeding programs. We hypothesized that a comprehensive analysis of the performance of these QC markers across ICRISAT‐ESA sorghum breeding lines will also give a fair reflection of their performance across the national programs, which predominantly derive their genetic stocks from ICRISAT.

## 2 MATERIALS AND METHODS

### 2.1 Plant material

We used 700 breeding lines from ICRISAT‐ESA breeding program classified based on the target trait (drought tolerant [DT] or tolerant to low temperature [LT]) or target agroecology (dry lowland [DL] or sub‐humid [SH] regions) (Table S1). Sixteen genotypes from Burkina Faso were included as an outgroup. All the breeding lines and Burkina Faso genotypes were planted in 300‐mL germination trays in a polyhouse at the World Agroforestry Centre (Nairobi, Kenya). For hybridity confirmation, parental lines (IESV240, KARIMTAMA1, F6YQ212, and FRAMIDA) and the putative F_1_s (IESV240 × F6YQ212; KARIMTAMA1 × FRAMIDA) were planted in 4‐L pots. Leaf sampling of breeding lines, parents of the crosses, and the putative F_1_ plants was done according to Intertek‐Agritech sampling protocol 14 days postemergence and dried at 50°C for 12–24 h. The dried leaf samples were sealed and shipped to the Intertek‐Agritech laboratory (Alnarp, Sweden) for DNA extraction and KASP genotyping. The breeding lines were genotyped using 49 KASP assays, 46 of which were informative. The KASP marker assays were available at Intertek‐Agritech through the Excellence in Breeding Platform (Table S1). Hybridity testing was done on a total of 39 F_1_ plants derived from crosses between IESV240 and F6YQ212 (18 crosses) and KARIMTAMA1 and FRAMIDA (21 crosses). The F_1_ plants alongside their parents were genotyped using 10 markers that were selected as follows: three SNPs from the top 10, three SNPs from those ranked between 11 and 20, and four trait markers that were already available at Intertek‐Agritech.

### 2.2 Statistical analysis

Genotypic data were analyzed in the R software environment (R Core Team, 2021). Preliminary quality assessment was performed using the snpReady package (Granato & Fritsche‐Neto, 2018) and entailed filtering the SNPs with a call rate cut‐off of ≥0.25, minor allele frequency (MAF) ≥ 0.01, and genotype missing data cut‐off of ≤0.4. Retained SNPs were used to evaluate the genetic diversity metrics of the genotypes, including polymorphism information content (PIC), MAF, Nei's genetic diversity (GD), and observed heterozygosity (*H*_o_). The markers were further ranked based on a combination of PIC, MAF, and *H*_o_, and the top 30 performers retained for further analysis.

**Core Ideas**

- Validated informative single nucleotide polymorphism markers should be deployed for routine use in African breeding programs.
- The use of quality control markers for routine hybridity confirmation will fast track varietal development process.
- The ease of using kompetitive allele‐specific PCR markers will enhance their utilization in sorghum breeding programs.

Given that these markers were developed specifically to be run under the Intertek facility, which is optimized to run 10 assays for every 376 samples, we compared the informativeness of the top 10, 20, and 30 assays for potential use as individual clusters of 10 or for combined analysis. Principal component analysis (PCA) was done to display the grouping of the genotypes using the factoextra package (Kassambara & Mundt, 2016). Using the *dist* function in R, a Euclidian distance matrix was generated that was used for hierarchical clustering and plotting of a dendrogram using the ward.D2 method. Discriminant analysis of principal components (DAPC) was used to establish the clustering pattern of the genotypes in the adegenet package (Jombart et al., 2010). K‐means clustering followed by assessment of the Bayesian information criterion (BIC) aided the inference of optimal number of clusters. The DLs, DT, and SH genotypes were separated and subjected to PCA and DAPC analysis using different combinations of SNPs.

## 3 RESULTS

### 3.1 Overall informativeness and chromosomal distribution of the QC SNP markers

Genotypes with more than 40% (127 genotypes) missing data were not considered. A majority (103 of 351) of genotypes bred for the DLs recorded missing data (Table S2). Three SNPs (snpSB00344, snpSB00356, and snpSB00367) were monomorphic and were therefore removed from downstream analysis. The performance of all the 46 polymorphic markers is included in Table S2. We have summarized the informativeness of all the markers and compared them with those of top 10, 20, and 30 markers in Table 1 for easy reference. We also removed the three SNPs (snpSB00343, snpSB00383, and snpSB00351) that reported *H*_o_ > 0.5 and analyzed the performance of the lowest 13 markers. Our ranking correctly classified superior‐performing markers among the top 10 that recorded the highest mean PIC and MAF with the corresponding lowest average *H*_o_ (Table 1). The lowest performing 13 markers were the least informative recording the lowest average PIC and MAF (Table 1).

**TABLE 1:** Summary of marker informativeness based on different categories.

Marker category	PIC		MAF		*H*_o_	
	Range	Mean	Range	Mean	Range	Mean
Top 10	0.36–0.37	0.37	0.39–0.49	0.43	0–0.05	0.02
Top 20	0.32–0.37	0.35	0.27–0.49	0.38	0–0.08	0.03
Ranked 11–20	0.32–0.36	0.34	0.27–0.38	0.33	0.01–0.05	0.03
Top 30	0.19–0.37	0.32	0.12–0.49	0.31	0–0.08	0.01
ALL markers (46)	0.02–0.37	0.27	0.01–0.5	0.25	0–0.84	0.08
Ranked 21–30	0.19–0.31	0.24	0.12–0.25	0.17	0.01–0.06	0.02
Lowest 13	0.02–0.31	0.13	0.01–0.25	0.08	0–0.48	0.07

Abbreviations: PIC, polymorphism information content; MAF, minor allele frequency; *H*_o_, observed heterozygosity.

The 46 markers were well distributed across the sorghum genome, except on chromosome 5, which had the highest number of markers at 13 (28%) (Table 2). The top 10 markers were localized in each of the 10 sorghum chromosomes except on chromosomes 2, 4, 7, and 8 (Table 2).

**TABLE 2:** The distribution of informative markers across the sorghum genome.

Chromosome	All markers (46)	Top 10	Top 20	Top 30
1	9	4	9	9
2	2	0	0	1
3	3	1	1	2
4	3	0	1	2
5	13	1	2	5
6	5	2	4	5
7	3	0	0	2
8	1	0	0	0
9	3	1	1	1
10	3	1	2	3

### 3.2 Population structure based on different marker clusters

**FIGURE 1:**
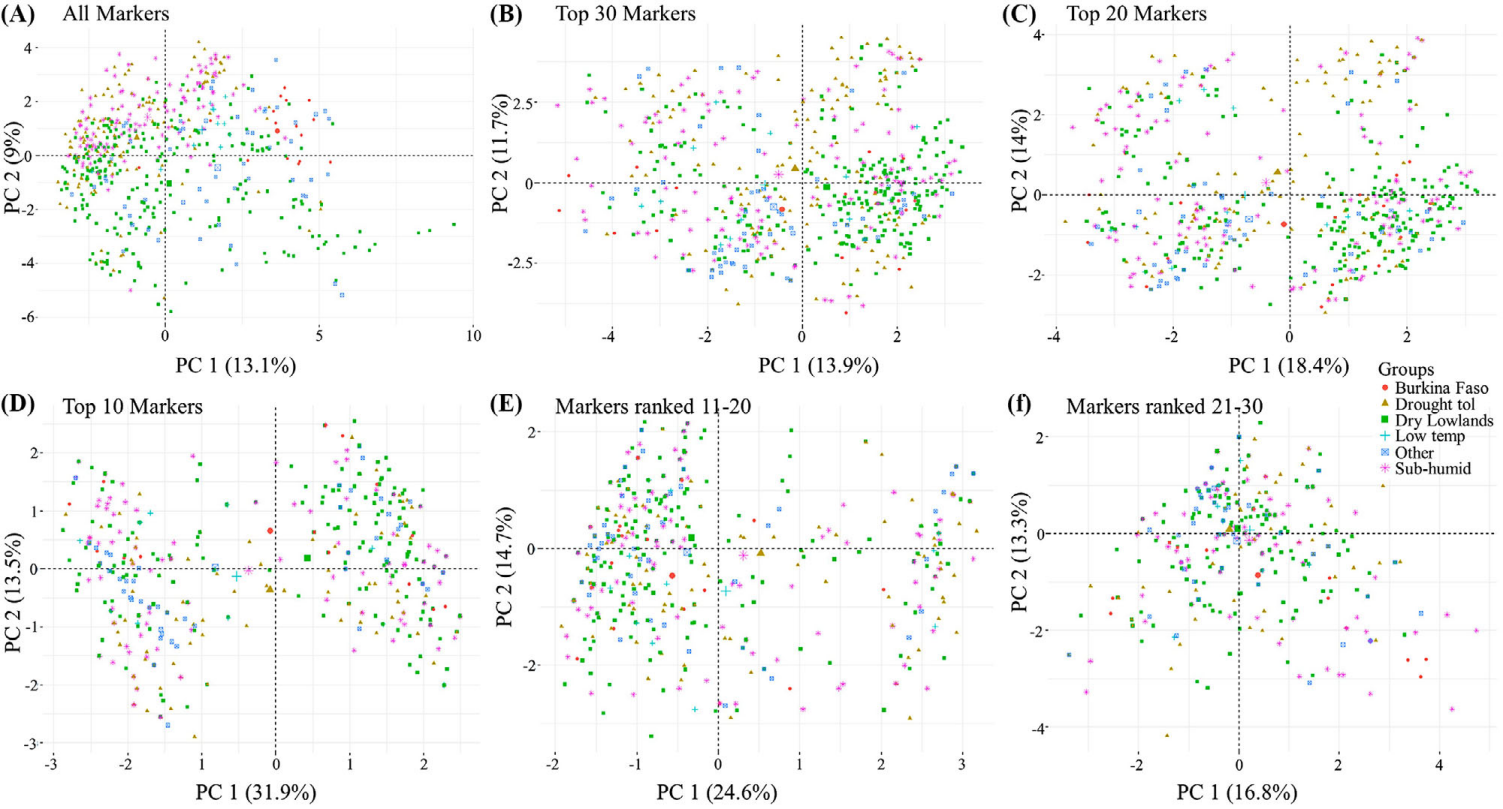
Principal component (PC) plots using different marker rankings scored across the entire germplasm set to show how informative they were. (A) All 46 markers resulted in the first 2 PCs explaining 22.1% genetic variation. (B) Top 30 markers resulted in the first 2 PCs explaining 25.6% genetic variation. (C) Top 20 most informative markers explained 32.4% genetic variation in the first 2 PCs. (D) Top 10 most informative markers explained 45.4% of genetic variation in the first 2 PCs. (E) Markers ranked 11–20 explained 39.3% of genetic variation in the first 2 PCs. (F) Markers ranked 21–30 explained 30.1% of genetic variation in the first 2 PCs.

We recorded a positive correlation between cluster PIC values and the genetic variation explained across the principal components (PCs) in each of the clusters. A high average PIC corresponded to higher genetic variation explained by the PCs (Figure 1). For example, the top 10 markers (mean PIC of 0.37) that explained 45% genetic variance within the first 2 PCs also reported a better separation of the breeding lines in comparison to the rest of the marker clusters (Figure 1D). The second‐best marker cluster was that of markers ranked from 11 to 20, which explained 39.3% variation in the first 2 PCs (Figure 1D). A combination of the top 20 markers explained 32.4% variation within the first PCs (Figure 1C).

**FIGURE 2:**
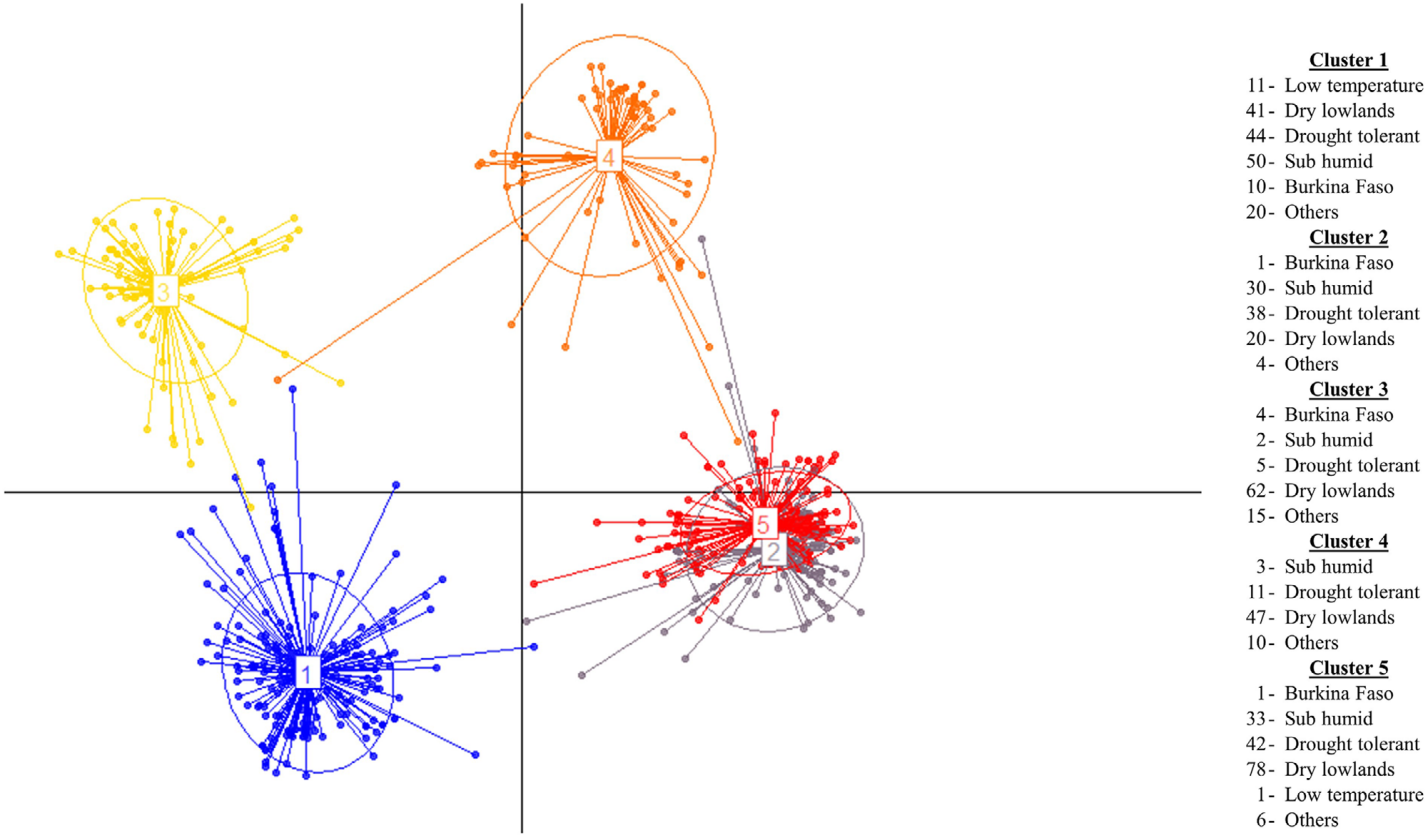
DAPC output revealing five groupings, of which three were distinct. Membership of each cluster is defined on the right‐hand side of the figure.

The DAPC done using top 30 markers distinguished the breeding lines into five major clusters, three of which were clearly distinct (clusters one, three, and four) (Figure 2). Table S1 provides detailed membership of the genotypes in their respective clusters. The LT breeding lines were predominantly in cluster 1, with just one additional LT genotype grouping in cluster 5 (Figure 2). DT and DL genotypes, on the other hand, were present in all the clusters in good numbers (except cluster 3 for DT), indicating the emphasis of DT trait ubiquity in the predominantly DL regions of ESA (Figure 2). Breeding lines targeting the SH regions were mostly present in clusters 1, 2 and 5. Burkina Faso breeding lines were present in all clusters except cluster 4.

### 3.3 Informativeness across Burkina Faso breeding lines

Out of the 49 markers tested across Burkina Faso (BF) genotypes, eight were monomorphic (snpSB00344, snpSB00348, snpSB00352, snpSB00356, snpSB00357, snpSB00358, snpSB00360, and snpSB00367) and two (snpSB00379 and snpSB00343) resulted in *H*_o_ > 0.5 and were therefore excluded from further analysis. The average performance of the remaining 39 markers across the BF breeding lines is summarized in Table 3. The markers were equally informative among the BF breeding lines despite using just 16 genotypes for the analysis, with PIC, MAF, and *H*_o_ ranges and mean being comparative to the larger datasets (Tables 1 and 3). Only two (snpSB00359 and snpSB00382) markers ranked among the top 10 performing markers in the larger dataset were ranked among the top 10 in their performance across the BF breeding lines (Table S3).

**TABLE 3:** The performance of markers across Burkina Faso's breeding lines.

Marker category	PIC		MAF		*H*_o_	
	Range	Mean	Range	Mean	Range	Mean
Top 10	0.35–0.37	0.36	0.33–0.47	0.39	0–0.19	0.05
Top 20	0.28–0.37	0.34	0.21–0.47	0.34	0–0.31	0.10
Ranked 11–20	0.28–0.36	0.32	0.21–0.38	0.29	0–0.31	0.15
Top 30	0.23–0.37	0.31	0.16–0.47	0.29	0–0.31	0.09
Ranked 31–39	0.06–0.21	0.17	0.03–0.14	0.10	0–0.12	0.03
All markers	0.06–0.37	0.28	0.03–0.47	0.25	0–0.31	0.07

Abbreviations: PIC, polymorphism information content; MAF, minor allele frequency; *H*_o_, observed heterozygosity.

### 3.4 Hybridity confirmation in F_1_ crosses

We used six markers (snpSB00320, snpSB00324, snpSB00364, snpSB00368, snpSB00369, and snpSB00387) alongside four other trait markers (data not shown) to test for hybridity among crosses. Three (snpSB00320, snpSB00324, and snpSB00368) out of six markers were polymorphic between IESV240 × F6YQ212 and were used to confirm hybridity of 16 out of 18 F1s tested (Table 4). The three markers also successfully revealed self‐pollinated plants that harbored the same alleles as the female parent across all the three polymorphic loci. Five (snpSB00320, snpSB00324, snpSB00364, snpSB00369, and snpSB00387) out of the six markers were polymorphic across the second cross between KARIMTAMA1 × FRAMIDA. However, only four SNPs (snpSB00320, snpSB00324, snpSB00369, and snpSB00387) could successfully confirm hybridity of 12 out of the 21 F1s tested (Table 4). snpSB00364 was not informative across the progenies. The four informative markers also revealed that the unsuccessful crosses (selfs) bore the same alleles as the female parent.

**TABLE 4:** Confirmation of F_1_ hybridity using developed SNP markers.

Sample	snpSB00320	snpSB00324	snpSB00364	snpSB00368	snpSB00369	snpSB00387	Hybridity confirmation
**IESV 240**	**T:T**	**G:G**	**G:G**	**T:T**	**G:G**	**T:T**	**Female parent**
**F6YQ212**	**C:C**	**C:C**	**G:G**	**C:C**	**G:G**	**T:T**	**Male parent**
IESV 240 × F6YQ212_1	T:C	C:G	G:G	C:T	G:G	T:T	Yes
IESV 240 × F6YQ212_18	T:C	C:G	G:G	C:T	G:G	T:T	Yes
IESV 240 × F6YQ212_2	T:C	C:G	G:G	C:T	G:G	T:T	Yes
IESV 240 × F6YQ212_3	T:C	C:G	G:G	C:T	G:G	T:T	Yes
IESV 240 × F6YQ212_4	T:C	C:G	G:G	C:T	G:G	T:T	Yes
IESV 240 × F6YQ212_5	T:C	C:G	G:G	C:T	G:G	T:T	Yes
IESV 240 × F6YQ212_6	T:C	C:G	G:G	C:T	G:G	T:T	Yes
IESV 240 × F6YQ212_7	T:C	C:G	G:G	C:T	G:G	T:T	Yes
IESV 240 × F6YQ212_9	T:C	C:G	G:G	C:T	G:G	T:T	Yes
IESV 240 × F6YQ212_10	T:C	C:G	G:G	C:T	G:G	T:T	Yes
IESV 240 × F6YQ212_11	T:C	C:G	G:G	C:T	G:G	T:T	Yes
IESV 240 × F6YQ212_12	T:C	C:G	G:G	C:T	G:G	T:T	Yes
IESV 240 × F6YQ212_13	T:C	C:G	G:G	C:T	G:G	T:T	Yes
IESV 240 × F6YQ212_14	T:C	C:G	G:G	C:T	G:G	T:T	Yes
IESV 240 × F6YQ212_15	T:C	C:G	G:G	C:T	G:G	T:T	Yes
IESV 240 × F6YQ212_16	T:C	C:G	G:G	C:T	G:G	T:T	Yes
IESV 240 × F6YQ212_8	T:T	G:G	G:G	T:T	G:G	T:T	No
IESV 240 × F6YQ212_17	T:T	G:G	G:G	T:T	G:G	T:T	No
**KARIMTAMA1**	**T:T**	**C:C**	**G:G**	**T:T**	**A:A**	**T:T**	**Female parent**
**FRAMIDA**	**C:C**	**G:G**	**A:A**	**T:T**	**G:G**	**C:C**	**Male parent**
KARIMTAMA1 × FRAMIDA_1	T:C	C:G	A:A	T:T	A:G	T:C	Yes
KARIMTAMA1 × FRAMIDA_2	T:C	C:G	A:A	T:T	A:G	T:C	Yes
KARIMTAMA1 × FRAMIDA_3	T:C	C:G	A:A	T:T	A:G	T:C	Yes
KARIMTAMA1 × FRAMIDA_4	T:C	C:G	A:A	T:T	A:G	T:C	Yes
KARIMTAMA1 × FRAMIDA_5	T:C	C:G	A:A	T:T	A:G	T:C	Yes
KARIMTAMA1 × FRAMIDA_6	T:C	C:G	A:A	T:T	A:G	T:C	Yes
KARIMTAMA1 × FRAMIDA_7	T:C	C:G	A:A	T:T	A:G	T:C	Yes
KARIMTAMA1 × FRAMIDA_8	T:C	C:G	A:A	T:T	A:G	T:C	Yes
KARIMTAMA1 × FRAMIDA_12	T:C	C:G	A:A	T:T	A:G	T:C	Yes
KARIMTAMA1 × FRAMIDA_18	T:C	C:G	A:A	T:T	A:G	T:C	Yes
KARIMTAMA1 × FRAMIDA_19	T:C	C:G	A:A	T:T	A:G	T:C	Yes
KARIMTAMA1 × FRAMIDA_17	T:C	C:G	A:A	T:T	Uncallable	T:C	Yes
KARIMTAMA1 × FRAMIDA_9	T:T	C:C	A:A	T:T	A:A	T:T	No
KARIMTAMA1 × FRAMIDA_10	T:T	C:C	A:A	T:T	A:A	T:T	No
KARIMTAMA1 × FRAMIDA_11	T:T	C:C	A:A	T:T	A:A	T:T	No
KARIMTAMA1 × FRAMIDA_13	T:T	C:C	A:A	T:T	A:A	T:T	No
KARIMTAMA1 × FRAMIDA_14	T:T	C:C	A:A	T:T	A:A	T:T	No
KARIMTAMA1 × FRAMIDA_15	T:T	C:C	A:A	T:T	A:A	T:T	No
KARIMTAMA1 × FRAMIDA_16	T:T	C:C	A:A	T:T	A:A	T:T	No
KARIMTAMA1 × FRAMIDA_20	T:T	C:C	A:A	T:T	A:A	T:T	No
KARIMTAMA1 × FRAMIDA_21	T:T	C:C	A:A	T:T	A:A	T:T	No

*Note*: Pink cells, female alleles; blue cells, male alleles; yellow cells, uninformative markers; green cells, successful crosses. The bold text signifies parent allele information.

## 4 DISCUSSION

We report in this study the successful validation of sorghum QC markers for routine application in breeding programs in ESA and beyond. We demonstrated the ability of the markers to distinguish ICRISAT breeding lines in ESA, select breeding lines from Burkina Faso's national breeding program, as well as their utilization in hybridity confirmation. We ranked the markers based on their performance to give users an easy time selecting the most informative depending on their resources and relevant application. While the validation used a specific germplasm set, we expect the markers to be equally informative across different germplasm collections globally.

A key consideration for the effective routine use of molecular markers in a breeding program is the cost of genotyping. Tremendous progress has been made in improving the ease and cost of SNP discovery using next‐generation sequencing. The markers used in the current analysis were made available through the KASP assay technology, which is easy to use and cost‐effective (Semagn et al., 2013; Smith & Maughan, 2014). The KASP assays are relatively easy to design using known SNP flanking sequences (He et al., 2014). Several global breeding programs have developed similar marker sets in different crops, including rice (*Oryza sativa* L.) (Gouda et al., 2021), maize (Qu et al., 2022), and sweetpotato [*Ipomoea batatas* (L.) Lam] (Gemenet et al., 2020), that are routinely being used by several programs under the coordination of the Excellence in Breeding Platform (https://excellenceinbreeding.org/). Easy access to sorghum QC markers will mean access to the establishment of a molecular breeding program, especially to many breeding programs in developing countries. The integration of molecular tools into a conventional breeding program is a significant step toward enhancing genetic gain (Gedil & Menkir, 2019).

The validated markers will be extremely useful to breeders for a myriad of genetic applications, such as characterization of genetic diversity, genetic relationship, population structure, genetic purity testing, early generation selection, hybridity testing, and parental verification (Ertiro et al., 2015). The use of the same marker set across different breeding programs will also enable ease of merging and/or comparing breeding lines. This will be especially important for sorghum breeding programs in Africa, where just a few breeding lines have been used and exchanged across several breeding programs despite the existence of a huge genetic diversity. A recent study using local Ethiopian sweet sorghum landraces reported an unexploited genetic resource (Disasa et al., 2016) that could be used to enhance the low yields in the continent (FAOSTAT, 2022) if the genetic relatedness is known.

While most studies have used PIC only to evaluate the discriminatory power of markers (Serrote et al., 2020), we used a combination of PIC, MAF, and Ho to rank the markers for their informativeness. We expected that the higher the MAF, the more powerful the discriminating ability of the SNP (Mammadov et al., 2010). We therefore discriminated against markers with lower MAF, especially because the markers were intended for broader use across several breeding programs. We also discriminated against SNP markers that recorded >20% heterozygosity and completely removed those that recorded >50% heterozygosity. Extremely high heterozygosity can be a result of technical difficulties in reading and interpreting the fluorescence signals or cross‐hybridization of the primers to homologous or homeologous genes (Würschum et al., 2013), therefore not desirable.

One of the major drawbacks in the African breeding programs is the lack of capacity to undertake rapid confirmation of successful crosses. The traditional hybridity confirmation process relies on morphological markers and is therefore time consuming and unreliable, yet it has been the only solution available for most programs in Africa. Although several studies have used KASP assays in sorghum for different purposes (Burow et al., 2019; da Silva et al., 2020; Sejake et al., 2021), this is the first time validated KASP assays will be made routinely and publicly accessible to breeders in Africa. Higher genetic gain has been reported in several crops (Biswas et al., 2023; Ibitoye et al., 2010; Xu et al., 2017) as a result of integrating genomic tools into breeding programs. It is expected that routine use of these molecular markers by breeders would result in improved breeding efficiency and consequent enhanced genetic gain in sorghum. However, the QC markers will need to be complemented with trait markers and other tools such as genomic selection (Goddard, 2009; Voss‐Fels et al., 2019) and speed breeding (Chiurugwi et al., 2019) if the full benefits of a modern breeding program were to be realized.

The distribution of the markers across various sorghum chromosomes was fair but could be improved. The over‐representation of markers on chromosomes 1 and 5 is not expected to have an impact on the performance of the markers as they are purely for QC. However, future QC marker development efforts should focus on closing the gaps to ensure the whole genome is evenly covered. Our results also revealed a fair distribution of the breeding lines across the five clusters generated from DAPC analysis. Full understanding of the extent of how representative the ESA breeding lines are in comparison to the global collection would require conducting a broader study that includes global representative sets such as the minicore (Upadhyaya et al., 2009), the GCP reference set (Billot et al., 2013), and the global diversity set (Casa et al., 2008). Several studies in sorghum suggest high genetic diversity (Cuevas & Prom, 2020; de Wet & Huckabay, 1967; Faye et al., 2019; Ganapathy et al., 2017; Harlan & de Wet, 1972; Maina et al., 2018), which should be reflected in a major breeding program such as that of ICRISAT–ESA.

It was important to use ICRISAT breeding lines for the initial validation of the markers, given the important role of ICRISAT in sharing breeding material with different breeding programs in Africa. We expect that most of these markers will be informative within and across most of the national programs in ESA. However, an initial validation from each national program will go a long way in prioritizing the most informative markers for their respective purposes. The superior performance of the markers across a random selection of 16 Burkina Faso breeding materials is indicative that the markers will be useful beyond ESA breeding programs.

Overall, the QC markers will go a long way in helping sorghum breeders to undertake routine genotyping and access quality data, irrespective of the genomic infrastructure available to them. Future activities should focus on providing training to breeders on how to utilize these tools in their breeding programs and increase efficiency of varietal development process. Future priorities should consider the validation of trait‐linked markers that would lead to improved precision in selection for traits of interest. Given the application of these markers to several breeding programs, the resourcing for such activities should be channeled through regional and global funding bodies. There will also be a need for better coordination to ensure that the experiences of different breeding programs are shared with the broader sorghum breeding community.

## AUTHOR CONTRIBUTIONS

**Davis M. Gimode**: Formal analysis; methodology; writing—original draft; writing—review and editing. **Grace Ochieng**: Methodology. **Santosh Deshpande**: Conceptualization; investigation. **Eric O. Manyasa**: Resources. **Clarisse P. Kondombo**: Resources. **Erick O. Mikwa**: Methodology. **Millicent O. Avosa**: Methodology. **Josephine Sarah Kunguni**: Methodology. **Kahiu Ngugi**: Resources. **Patrick Sheunda**: Resources. **McDonald Bright Jumbo**: Resources. **Damaris A. Odeny**: Conceptualization; formal analysis; funding acquisition; methodology; project administration; supervision; writing—original draft; writing—review and editing.

## CONFLICT OF INTEREST STATEMENT

The authors declare no conflicts of interest.

## Supporting information


**Supplementary Table 1**. Details of the 716 ICRISAT‐ESA breeding lines that were characterized.
**Supplementary Table 2**. The ranking of 46 SNPs used to genotype ICRISAT‐ESA and select Burkina Faso sorghum breeding lines.
**Supplementary Table 3**. The 39 SNPs that were informative across the Burkina Faso breeding lines.

## Data Availability

All data generated or analyzed during this study are included in this published article and/or made publicly available through the Excellence in Breeding Platform website: https://excellenceinbreeding.org/.
